# The impact of diurnal variations on emergence delirium following general anesthesia and surgery in children

**DOI:** 10.3389/fped.2024.1437460

**Published:** 2024-10-16

**Authors:** Wei Wei, Haihang Xie, Yingyi Xu, Jingwen Qin, Xinying Guo, Xingrong Song, Gaofeng Yu, Na Zhang, Daqing Ma, Yonghong Tan, Tianyun Zhao

**Affiliations:** ^1^ Department of Anesthesiology, Guangzhou Women and Children’s Medical Center, Guangzhou Medical University, Guangzhou, China; ^2^ Division of Anaesthetics, Pain Medicine & Intensive Care, Department of Surgery & Cancer, Faculty of Medicine, Imperial College London, Chelsea and Westminster Hospital, London, United Kingdom

**Keywords:** emergence delirium, diurnal variations, circadian rhythms, pediatric, general anesthesia

## Abstract

**Background:**

Emergence delirium (ED) is a widely recognized issue that prolongs mechanical ventilation and post-anesthesia care unit (PACU) resuscitation time, consequently increasing hospital costs and mortality. Postoperative disturbance in circadian rhythms, commonly leading to sleep disorders, has been identified as a significant risk factor for ED. However, the influence of surgery timing (morning vs. afternoon) on the incidence of ED in pediatric patients undergoing general anesthesia remains unknown.

**Methods:**

Patients aged 2–6 years who were operated on under general anesthesia with a bispectral index value between 50 and 60 were categorized based on anesthesia start time into either the morning surgery group (Group M, 8:00–12:00) or the afternoon surgery group (Group A, 13:00–17:00). The primary outcome was the post-extubation incidence of ED assessed by the Cornell Assessment of Pediatric Delirium (CAPD) score. Secondary outcomes included extubation time, duration of PACU stay, and adverse postoperative events and complications.

**Results:**

We recruited a total of 560 patients, 280 in group M and 280 in group A. Compared to Group M, Group A exhibited a significantly higher incidence of ED (*p* < 0.001), elevated CAPD scores (*p* < 0.001), and prolonged PACU stays (*p* < 0.001). Notably, there was no significant difference in extubation time and anesthesia-related adverse events or other postoperative complications between the groups.

**Conclusion:**

Our study highlights that the time of surgery significantly affects the incidence of ED, CAPD scores, and PACU stay duration in children. Further validation of these findings may guide future strategies to reduce ED.

## Introduction

Emergence delirium (ED) is a complex post-anesthetic perceptual disorder and psychomotor agitation characterized by purposeless resistance movements and is particularly prevalent in children of preschool age following a sevoflurane-based anesthetic (1, 2). Although the incidence of pediatric ED varies, it can be notably high, ranging between 10% and 80% (3). The underlying mechanisms are largely unknown yet identified risk factors of ED include surgery type, age, anesthesia type, preoperative anxiety, preexisting pathological status, and pain (4–6). In general, the ED condition is transient and reversible; however, it is associated with postoperative behavioral issues (7). Children who experience postoperative delirium are at high risk of developing new maladaptive behaviors compared with those without such complications (8). Therefore, identifying ED-related factors and reducing their incidence is important during the perioperative period for young patients.

Circadian rhythms, which are orchestrated by the suprachiasmatic nucleus (SCN) of the hypothalamus, govern our daily physiological and behavioral cycles (9). These rhythms are influenced by various exogenous factors, including light exposure and melatonin secretion (10, 11). Furthermore, the timing of surgery procedures and anesthesia has been shown to exert a significant influence on perioperative circadian rhythms (12–14). Notably, surgeries conducted in the morning, as opposed to those in the afternoon, have been associated with greater disruption of circadian rhythms and sleep patterns in elderly patients, consequently elevating the risk of postoperative delirium, particularly when general anesthesia is administered (15, 16). Despite the widespread use of general anesthesia in pediatric surgeries, the impact of surgery timing on the incidence of ED in children remains insufficiently explored. This observational clinical study aimed to elucidate whether the timing of general anesthesia (morning vs. afternoon) influences the incidence of ED in children, thereby addressing a critical gap in the existing literature.

## Materials and methods

### Ethical approval and registration

This prospective observational single-center cohort study was conducted in the post-anesthesia care unit (PACU) of Guangzhou Women and Children's Medical Center from May 2022 to May 2023. The study protocol was approved by the ethics committee of the Guangzhou Women and Children's Medical Center [(2022)119A01] and registered with the Chinese Clinical Trials Registry (ChiCTR2200065441). Written consent from all participants’ parents or legal guardians was obtained before the study enrollment.

### Patients

Patients who received surgery (including abdominal, head and face, urological, and orthopedic surgeries) under general anesthesia were enrolled in this study. The inclusion criteria were preschool children aged 2–6 years with a physical status graded as I or II according to the American Society of Anesthesia (ASA) classification of physical status who were admitted to the PACU after surgery under general anesthesia. Exclusion criteria were as follows: (1) children undergoing cranial surgery; (2) children with a history of ischemic hypoxic encephalopathy in the past; (3) children with generalized developmental disorders; (4) children with developmental delay or neurological disorders, an abnormal airway such as obstructive sleep apnea, or a history of sedative or psychotropic medications; (5) children with a physical status graded ≥III according to the ASA classification who were transferred to the ICU after surgery; and (6) children whose parents or legal guardians did not understand and agree to the content of the informed consent form.

### Perioperative management

All patients received intravenous access the night before surgery, were not premedicated, and fasted for 8, 4, and 2 h for solids, breast milk, and clear fluids, respectively. The participants were divided into the morning group (Group M, 8:00–12:00) and the afternoon group (Group A, 13:00–17:00) based on the start time of the anesthesia. Upon arrival in the operating room, an electrocardiogram, and non-invasive arterial pressure and pulse oxygen saturation monitoring (SpO_2_) were applied and monitored at 5-min intervals throughout the surgery. End-tidal carbon dioxide and body temperature were monitored after induction, with levels maintained between 35 and 45 mmHg for carbon dioxide and 36°C–37°C for temperature. Anesthesia was induced with phencyclidine hydrochloride (0.01 mg/kg; Nhwa, Jiangsu, China), sufentanil (0.2 μg/kg; Humanwell Healthcare, Hubei, China), cis-atracurium (0.15 mg/kg; Hengrui, Jiangsu, China), and propofol (3 mg/kg; Kelun, Sichuan, China). After laryngeal mask placement or endotracheal intubation, anesthesia was maintained by inhaled sevoflurane (1%–3%) in 50% oxygen, along with intermittent intravenous administration of sufentanil and cis-atracurium. The bispectral index (BIS; Covidien IIc, MA, USA), which has been widely validated in children and recommends a range of 40–60 as in adults (17), was used to monitor the depth of anesthesia. In our study, the BIS levels were maintained between 50 and 60. For urological and abdominal surgeries, an ultrasound-guided transversus abdominis plane (TAP) block with 0.2% ropivacaine at a dose of 1 ml/kg was administered after induction as part of a combined anesthesia approach. After surgery, patients were immediately transferred to the PACU for extubation and recovery in our medical center. In the PACU, anesthesiologists performed extubation once the patients began breathing spontaneously with a tidal volume of at least 6 ml/kg and were able to open their eyes either spontaneously or in response to verbal commands. A team member, the chief anesthesiologist in the PACU, assessed ED using the Cornell Assessment of Pediatric Delirium (CAPD) and evaluated pain intensity with the Face, Legs, Activity, Cry, and Consolability (FLACC) scale 1 min after extubation and every 5 min thereafter. The highest recorded scores during the PACU stay were used for the evaluation. Children received propofol (1 mg/kg) for the management of ED, and sufentanil (0.1 μg/kg) was administered if their FLACC score was ≥4 in the PACU. Patients were discharged from the PACU when their Aldrete score reached 9 or above provided that they showed no signs of agitation or pain.

### Blinding

The researchers who conducted the preoperative interviews, including history collection, and physical and cognitive assessments, were blinded to the procedure and anesthesia assignments. The two physicians responsible for assessing postoperative delirium were trained to achieve inter-rater agreement above 75% and were also blinded to the study group assignments.

### Study endpoints

The primary outcome was the incidence of ED (during PACU stay) assessed by the CAPD score after extubation. Secondary outcomes included the extubation time, duration of PACU stay (from PACU admission to PACU discharge), the intensity of pain assessed by the FLACC scale, the proportion of patients required rescue propofol for ED during the PACU stay, postoperative adverse events, and other anesthetic-related complications such as vomiting, laryngospasm, and hypoxia (defined as a SpO_2_ level < 92% maintained for more than 3 min). The duration of monitoring includes the entire PACU stay. CAPD, an adaptation of pediatric anesthesia emergence delirium (PAED), was designed to capture all delirium subtypes, specifically the hypoactive phenotype, and can be used in children of all ages while PAED is applicable to patients aged from 19 months to 6 years (18). The accuracy and efficacy of the Chinese version of CAPD have been validated in pediatric surgical patients with optimal sensitivity (87%) and specificity (98%) when the cut-off point was set at 9 (19). The FLACC score, a validated behavioral observation scale for assessing pain in infants and children aged 2 months to 7 years, assesses pain by rating five behaviors (facial expression, leg position, degree of activity, quality of cry, and consolability) on a scale of 0 to 2 (20). The total score ranges from 0 to 10, with a higher score indicating more severe pain.

### Sample size estimation

According to the results of our preliminary study before this study, the incidence of ED was 36.4% (SD 5.1%) in morning surgery and 44.3% (SD 11.9%) in afternoon surgery in our operation center. Assuming a category I error of 0.05 and a shedding rate of 10%, 560 patients were selected as the sample size target with 280 patients in Group M and 280 patients in Group A.

### Statistical analysis

GraphPad Prism 8.0 software and SPSS 20.0 statistical software (SPSS, Chicago, IL, USA) were used for the data analysis. Continuous data were represented as mean (SD) and were analyzed by Student's independent-samples *t*-test and the Mann–Whitney *U*-test. The chi-square test was used to analyze differences in the categorical data. A *p*-value < 0.05 was considered significant. We employed a multivariable logistic regression model to explore the association between surgery timing variations and ED. Confounding factors identified in the literature as potential influencers of ED, such as age, type and duration of surgery, history of anxiety, and proportion of TAP, were included as covariates. The confounders were selected based on their statistical significance in the univariable logistic regression analysis, which was used to identify any potential associations between these variables and ED.

## Results

### Baseline characteristics

**Figure 1:**
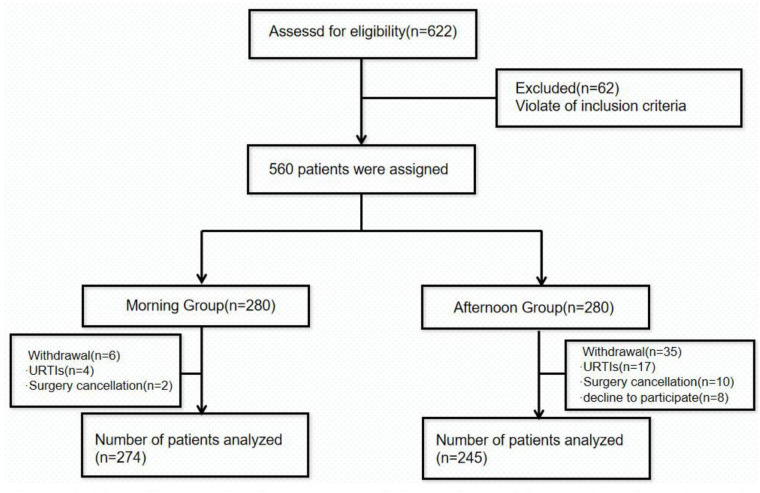
Flow diagram showing the progress of the study participants throughout the study. URTIs, upper respiratory tract infections.

We initially included 622 patients and assessed their eligibility for this study (Figure 1). Of these, 62 were excluded due to not meeting the inclusion criteria. Thus, 560 eligible patients were enrolled in the study and they were assigned to either the morning group (*n* = 280) or the afternoon group (*n* = 280) according to the time of anesthesia induction. However, due to further exclusions primarily due to upper respiratory tract infections (21 children) and abnormal laboratory results (12 children), the final analysis included 274 patients in the morning group and 245 patients in the afternoon group. Overall, the two groups were well-matched for baseline variables, including age, weight, height, BMI, sex, anesthesia choice (laryngeal mask or endotracheal intubation), and the use of nerve blocks (*P* > 0.05; Table 1).

**Table 1:** Demographics and perioperative variables.

	Morning group (*n* = 274)	Afternoon group (*n* = 245)	*P*-value
Age (months)	54.5 (15.1)	56.2 (14.9)	0.19
Weight (kg)	17.7 (8.6)	17.4 (3.8)	0.47
Height (m)	1.1 (0.1)	1.1 (0.1)	0.59
BMI (kg m^−2^)	15.1 (9.6)	14.9 (2.2)	0.69
Sex, *n* (%)			0.83
Male	199 (72.6%)	180 (73.5%)	
Female	75 (27.4%)	65 (26.5%)	
Anesthesia, *n* (%)			0.35
GA with endotracheal intubation	195 (71.2%)	165 (67.3%)	
General anesthesia with laryngeal mask	79 (28.8%)	80 (32.7%)	
TAP, *n* (%)			0.08
Yes	66 (24.1)	76 (31)	
No	208 (75.9)	169 (69)	

BMI, body mass index; GA, general anesthesia, TAP, transversus abdominis plane block.

Data are presented as mean (SD) or number (%).

### Study outcomes

As shown in Table 2, the incidence of ED was significantly higher in Group A compared to Group M (38.4% vs. 12%, *P* < 0.001, Table 2). Group A also had higher CAPD scores [7.5 (6.5) vs. 3.4 (4.6); *P* < 0.001, Table 2] and a greater need for rescue propofol during their PACU stay (12.8% vs. 39.6%, *P* < 0.001, Table 2). However, the postoperative extubation times were similar, with no statistically significant difference between the groups [27.4 (13.6) min vs. 25.8 (14.5) min; *P* = 0.21, Table 2]. Notably, the duration of PACU stay was significantly longer in Group A compared to Group M [45.1 (17.5) min vs. 52.4 (17.3) min; *P* < 0.001, Table 2]. There was no statistically significant difference in the use of rescue analgesics (sufentanil) or the occurrence of postoperative adverse events (Table 2). A multivariable logistic regression analysis showed that surgery timing (OR 4.966, 95% CI 3.129–7.978, *P* < 0.001), surgery type (OR 2.085, 95% CI 1.026–4.238, *P* < 0.01), and sex (OR 0.378, 95% CI 0.213–0.671, *P* < 0.01) (Table 3) were associated with an increased risk of ED. A significantly reduced incidence of ED in Group M remained after adjusting for these confounders.

**Table 2:** Effectiveness outcomes and postoperative complications.

	Morning group (*n* = 274)	Afternoon group (*n* = 245)	*P*-value
Incidence of ED, *n* (%)	33 (12.0%)	94 (38.4%)	<0.001
CAPD score	3.4 (4.6)	7.5 (6.5)	<0.001
Incidence of propofol rescue, *n* (%)	35 (12.8%)	97 (39.6%)	<0.001
Incidence of sufentanil rescue, *n* (%)	12 (4.4%)	17 (6.9%)	0.21
Extubation time (min)	25.8 (14.5)	27.3 (13.6)	0.21
PACU time (min)	45.1 (17.5)	52.4 (17.3)	<0.001
Postoperative complications			
Hypoxia	0	0	NA
PONV, *n* (%)	61 (22.3%)	68 (27.8%)	0.15
Laryngospasm	1	0	NA
All-cause 30-day mortality	0	0	NA

CAPD, Cornell Assessment of Pediatric Delirium; ED, emergence delirium; PACU, post-anesthesia care unit; PONV, postoperative nausea and vomiting.

Data are presented as mean ± SD or number (%). Hypoxia was defined as SpO_2_ < 92% maintained for more than 3 min.

**Table 3:** Relationship between the characteristics of the patients and the presence of ED.

Variables	Univariate analysis		Multivariate analysis	
	*P*	OR (95% Cl)	*P*	OR (95% Cl)
Age	**0** **.** **005**	**1.018** (**1.005**–**1.031)**	—	
Weight	0.679	0.282 (0.047–1.959)	—	
Height	**0**.**001**	**1.093** (**1.038**–**1.150)**	—	
BMI	0.249	1.053 (0.965–1.149)	—	
Sex	**0**.**001**	**0.394** (**0.231**–**0.671)**	**0.001**	**0.378** (**0.213**–**0.671)**
Surgery type	**0**.**000**	**3.561** (**2.350**–**5.397)**	**0.042**	**2.085** (**1.026**–**4.238)**
Surgery timing	**0**.**000**	**4.546** (**2.911**–**7.099)**	**0.000**	**4.966** (**3.129**–**7.978)**
Anesthesia type	0.104	0.704 (0.461–1.075)	—	
Nerve block	0.331	1.243 (0.802–1.929)	—	

CI, confidence interval; OR, odds ratio.

Data are presented as the relationship (indicated by the odds ratio) between the number of patients who experienced emergence delirium and the following variables: nerve block, anesthesia type, surgery timing, surgery type, BMI, weight, height, age, and sex.

The bold values was considered significant.

## Discussion

In this observational study, we found a significantly higher incidence of ED, elevated total CAPD scores, and increased need for rescue sedatives in the afternoon surgery group compared to the morning group. In addition, afternoon surgeries were associated with longer stays in the PACU. Therefore, our results suggest that conducting surgery in the morning may lead to a lower incidence of ED.

ED is one of the most common complications in pediatric anesthesia, as noted in the evidence-based guidelines from the European Society of Anaesthesiology and Intensive Care. These guidelines underscore various risk factors that contribute to pediatric ED, including preoperative anxiety, age, pain, medications (including inhalation of anesthetics/sedatives, opioids, and anticholinergic agents), and surgical methods (21). After adjusting for these confounders, a significantly reduced incidence of ED was observed in Group M, suggesting that circadian rhythm may serve as an additional risk factor for ED in this population. Previous studies have demonstrated that surgery and anesthesia could cause disturbances in sleep, which is regulated by circadian rhythms (22). For example, Yang et al. (15) reported that morning surgery was associated with fewer sleep disturbances in elderly patients. However, limited research has investigated whether perioperative sleep/circadian rhythm disruption affects the incidence of ED in children. Although our results indicate that morning surgeries significantly decrease the occurrence of ED in children, we did not assess perioperative sleep quality in this observational study. Therefore, further research is needed to determine whether this reduction is due to sleep/circadian rhythm disruption.

Moreover, the interaction between circadian rhythms and general anesthetics may be another important mechanism underlying daytime variation of ED (23). Sevoflurane, one of the most commonly used volatile anesthetics, is one of the proposed risk factors for ED, especially in children (6). Several animal studies have demonstrated that general anesthetics disturb circadian rhythms at multiple levels, suggesting that anesthesia contributes to postoperative circadian disruption and recovery. For example, Kadota et al. (24) found that sevoflurane has a time-dependent effect on the expression of mPer2, the core clock gene in the SCN, with daytime anesthesia having the greatest inhibitory effect on mPer2 expression. Moreover, the expression of mPer2 in the SCN decreases significantly after anesthesia, with normalization after 24 h (25). In addition, circadian rhythms also have an impact on the pharmacodynamics and pharmacokinetics of intravenous anesthetics. It has been suggested that relatively large amounts of anesthetics are required in the morning, which may be related to faster liver and kidney metabolism during that time (13). In animal experiments, the pharmacological effects of propofol can exhibit up to a threefold variation when administered at different times (26). In this study, we also found that compared with the morning surgery group, a higher proportion of children in the afternoon surgery group required intravenous anesthetics in the PACU for postoperative delirium rescue (12.4% vs. 31%).

In addition to anesthetic-related factors, circadian rhythms also exert a regulating effect on the physiological status of children. In this study, we observed a significant difference in the duration of PACU stays between the afternoon group and the morning group, with the former exhibiting a significantly longer PACU stay [52.4 (17.3) min compared to 45.1 (17.5) min in the latter]. First, we postulate that as children often need naps during the noon period, scheduling surgery in the afternoon may disrupt their biological clocks and potentially impact their recovery. In addition, children undergoing surgery in the afternoon may be more susceptible to various physiological challenges, such as an electrolyte imbalance, hypotension, local tissue ischemia/hypoxia, and other symptoms due to a longer fasting time and insufficient preoperative fluid replacement. Such physiological disturbances could lead to delayed awakening and decreased awakening quality. Moreover, Hou et al. (27) found that the level of cortisol in the afternoon surgery group was higher than that in the morning surgery group, suggesting that afternoon surgery may trigger a more pronounced stress response in children, further influencing their post-anesthetic awakening process.

Although our pragmatic, prospective study has many advantages, including a substantial sample size of 560 surgical patients which enabled us to detect significant differences in the primary and some secondary endpoints between the groups, it is important to acknowledge several limitations inherent in our study design: (1) Our study exclusively focused on morning and afternoon groups, omitting an evening group from consideration. This choice was primarily dictated by the fact that nighttime surgeries in our hospital are predominantly emergency procedures, characterized by the patient’s preoperative condition often involving pain, hypoxia, and excessive tension that are uncommon for daytime surgeries. (2) Our study did not incorporate the measurement of dim-light melatonin onset (DLMO), a biomarker for the internal circadian rhythm (28), and did not monitor postoperative changes in body temperature, which exhibits rhythmicity. (3) The inclusion criteria did not consider whether participants had sleep disturbances or circadian rhythm disorders before surgery. These factors could potentially confound the observed effects of circadian rhythms on postoperative outcomes (29). (4) We only collected the perioperative parameters, and we acknowledge the possibility of long-term effects on cognitive function and sleep quality that may be associated with the immediate and reversible circadian rhythm disruption after surgery.

## Conclusion

Our study suggests that daytime variation (morning and afternoon) in surgery timing may have an important effect on the incidence of postoperative delirium in children who undergo general anesthesia. Specifically, children scheduled for afternoon surgeries exhibited notably higher rates of delirium, coupled with prolonged stays in the PACU, compared to their counterparts who underwent morning surgeries. This difference may be related to an interaction between circadian rhythms, general anesthetics, and/or surgery and needs further study.

## Data Availability

The datasets presented in this study can be found in online repositories. The names of the repository/repositories and accession number(s) can be found in the article/Supplementary Material.
